# First application of carbon dioxide digital subtraction enterography for stricture evaluation in Crohn’s disease

**DOI:** 10.1055/a-2739-2280

**Published:** 2025-11-21

**Authors:** Akihiro Maruyama, Kohei Takano, Junya Yamada, Hiroki Kato, Sakurako Isobe, Makoto Kobayashi

**Affiliations:** 1 37036 Department of Gastroenterology, Yokkaichi Municipal Hospital, Yokkaichi, Japan; 2 36590 Department of Gastroenterology, Nagoya University Hospital, Nagoya, Japan

Digital subtraction imaging (DSI) is a radiological technique used to enhance the visualization of anatomical structures. It subtracts a pre-contrast image from a post-contrast image, thereby effectively eliminating background noise. This method is commonly used in angiography and interventional radiology 1 , and recent reports have also suggested its utility in gastrointestinal imaging 2 3 4 . We applied CO _2_ digital subtraction enterography (CDDSE) as a novel approach during double-balloon endoscopy.

A 57-year-old man with a 10-year history of Crohn’s disease, notable for poor treatment adherence and irregular follow-up, presented with a 1-month history of recurrent nausea and progressive abdominal distension. Abdominal computed tomography demonstrated wall thickening at the terminal ileum. A transanal double-balloon endoscopy was performed using an EN-580T (FUJIFILM, Tokyo, Japan) equipped with a Cast Hood (TOP, Tokyo, Japan) at its tip. Conscious sedation was achieved with intravenous midazolam, and an antispasmodic agent, hyoscine butylbromide, was administered. DSI was conducted with the Ultimax-i DREX-U180 fluoroscopy system (Canon, Tokyo, Japan). CDDSE was then performed, which enabled evaluation of the stricture and the bowel up to the previously placed clip marking the maximal oral reach ( Video 1 and Fig. 1 ).

**Fig. 1:**
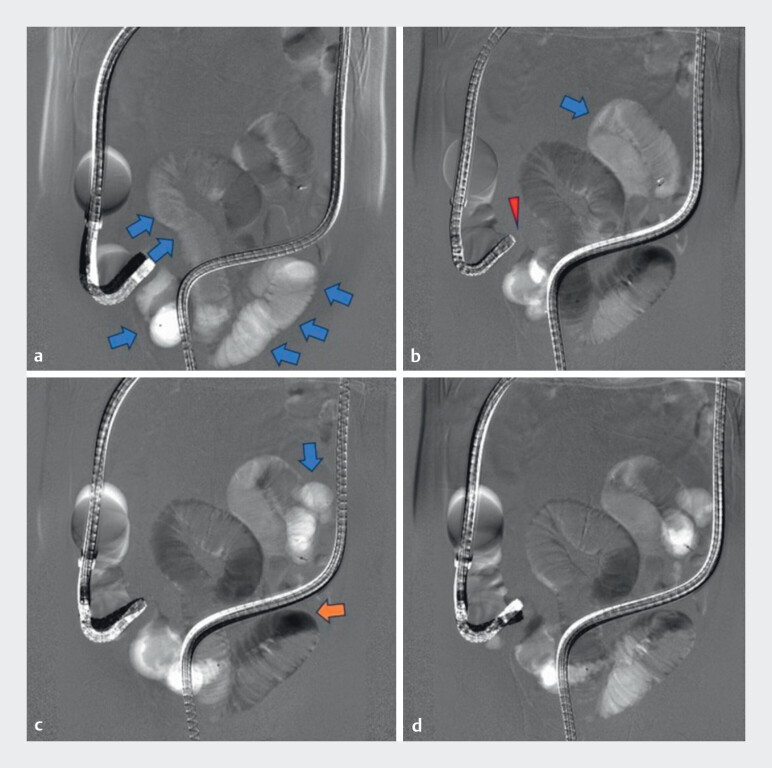
Panels ( **a–d** ) show serial CDDSE digital subtraction images. Compared with the pre-image, areas with greater CO _2_ accumulation appear white (blue arrows), whereas those with less CO _2_ appear black (orange arrows), reflecting CO _2_ flow and enabling assessment of the intestine. The stricture is visualized as indicated by the red arrowhead.

Carbon dioxide digital subtraction enterography (CDDSE) performed during double-balloon endoscopy provided clear delineation of an ileal stricture in Crohn’s disease, demonstrating feasibility and procedural simplicity.Video 1

No adverse events occurred during the procedure. The total procedure time from endoscope insertion to balloon dilation was 31 minutes, and the cumulative duration of the two CDDSE acquisitions was 30 seconds. The total radiation dose was 27.3 mGy, of which 4.9 mGy was attributable to the two CDDSEs. Balloon dilation was subsequently performed ( Fig. 2 ), leading to prompt improvement of the patient’s clinical symptoms.

**Fig. 2:**
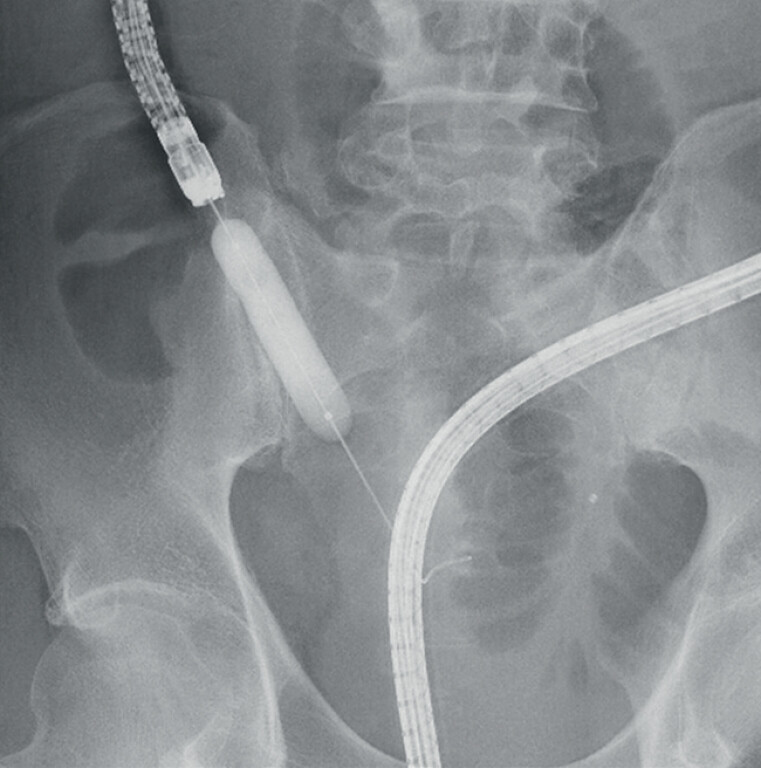
Balloon dilation was performed using a 15-mm balloon dilator, maintained for 1 minute.

As the technique requires only pressing the endoscopic insufflation button in synchrony with DSI acquisition, it enables clear delineation of intestinal strictures in a short time and with great procedural simplicity. Because CO _2_ flows more readily than liquid contrast agents, CDDSE may enable visualization of longer intestinal segments within shorter acquisition times.

Endoscopy_UCTN_Code_TTT_1AP_2AD

## Notes

Endoscopy E-Videos https://eref.thieme.de/e-videos
*E-Videos* is an open access online section of the journal *Endoscopy* , reporting on interesting cases and new techniques in gastroenterological endoscopy. All papers include a high-quality video and are published with a Creative Commons CC-BY license. Endoscopy E-Videos qualify for HINARI discounts and waivers and eligibility is automatically checked during the submission process. We grant 100% waivers to articles whose corresponding authors are based in Group A countries and 50% waivers to those who are based in Group B countries as classified by Research4Life (see: https://www.research4life.org/access/eligibility/ ). This section has its own submission website at https://mc.manuscriptcentral.com/e-videos .
